# Identification of constituent herbs in ginseng decoctions by DNA markers

**DOI:** 10.1186/s13020-015-0029-x

**Published:** 2015-01-30

**Authors:** Yat-Tung Lo, Ming Li, Pang-Chui Shaw

**Affiliations:** School of Life Sciences, The Chinese University of Hong Kong, S.A.R N.T, Shatin, Hong Kong China; State Key Laboratory of Phytochemistry and Plant Resources in West China (CUHK), Institute of Chinese Medicine, The Chinese University of Hong Kong, Shatin, N.T., Hong Kong China

**Keywords:** Ginseng, Decoction, Molecular authentication, DNA marker, Multiplex PCR, DNA sequencing

## Abstract

**Background:**

DNA in herbal decoctions is usually fragmented by extensive boiling and is usually regarded as unsuitable for molecular authentication. This study aims to evaluate the feasibility for molecular authentication methods by multiplex polymerase chain reaction (PCR) and DNA sequencing for decoctions.

**Methods:**

The DNA extraction procedure, sample pulverization and boiling time were examined in (1) single herb decoction with *Panax ginseng* (“ginseng”) or *P. quinquefolius* (“American ginseng”), (2) decoctions of two classical ginseng prescriptions of five herbal materials (*Aconitum carmichaeli*, *Atractylodes macrocephala*, *P. ginseng*, *Glycyrrhiza uralensis* and *Zingiber officinale*) and (3) a commercial ready-to-serve ginseng soup. Primers were designed from 26S-18S, ITS2 and trnH-psbA region, with DNA sequences obtained from GenBank. Multiplex PCR was also employed in ginseng or American ginseng decoctions to differentiate between these two herbs.

**Results:**

All six herbal species tested in this study could be identified in decoctions. We had four main observations: (1) sample pulverization before boiling improved PCR detection; (2) prolonged boiling increased the DNA concentration but decreased the intactness of DNA fragments; (3) ginseng could be differentiated from American ginseng by multiplex PCR; (4) identification of individual herbs in multi-herb decoctions with prolonged boiling time of 180 min could be achieved.

**Conclusions:**

DNA could be amplified from extensively boiled ginseng decoctions, multi-herb decoctions and commercial soup although DNA degradation was critical to successful PCR.

**Electronic supplementary material:**

The online version of this article (doi:10.1186/s13020-015-0029-x) contains supplementary material, which is available to authorized users.

## Background

Intentional or inadvertent adulteration of herbal products, including health food and dietary supplements, can cause adverse effects or even poisoning [1-6]. For instance, misidentification of the toxic vine *Gelsemium elegans* as *Mussaenda pubescens* (an ingredient of Chinese health drinks) caused two poisoning cases in Hong Kong [3]. Adulteration of the anti-inflammatory agent *Stephania tetrandra* by the toxic herb *Aristolochia fangchi* led to more than 100 cases of kidney failure and urothelial carcinoma in Belgium [4]. Adulterants have also been found in expensive herbs such as *Panax quinquefolius* [7], and materials from endangered species such as the horn of *Saiga tatarica* [8]. Therefore, reliable and effective methods of authentication are crucial for the quality control of herbal materials and their products.

Authentication of highly processed herbal materials is difficult. Examples include comparison of the morphological and microscopic characteristics of easily confused herbs and their dregs after boiling [9,10]. The approaches described could be useful, but the dregs in a decoction are usually discarded before consumption. Authentication is also possible by chemical or physical analyses such as high performance liquid chromatography [11], mass spectrometry [12] and nuclear magnetic resonance [13]. However, the chemical constituents in decoction may be altered by physiological conditions, harvesting period, storage and boiling conditions of the herbs involved. Such methods also make differentiation between related species quite difficult owing to their similar chemical profiles and demanding sophisticated instrumentation [14].

Since the 1990s, molecular methods have been used for authentication. There have been reports of using internal transcribed spacer 2 (ITS2) region to distinguish *Lycium barbarum* from its various types [15]; polymerase chain reaction (PCR) fragment length polymorphisms in the 3′ untranslated region of cDNA sequences to identify the root of *Astragalus membranaceus* [16]; DNA microarrays to authenticate ginseng drugs [17]; and PCR based method to characterize alfalfa and red clover [18]. Molecular methods and employment of DNA barcodes can produce accurate, reliable and sensitive results [19,20].

China was the first country to recognize molecular methods as a legal basis for authentication of crude herbal drugs, including *Fritillaria unibracteata* [21] and *Zaocys dhumnades* [22]. The future of molecular authentication is promising, even with DNA degradation during processing. For instance, molecular authentication of meats in processed food had been reported [23-25] and short microsatellite DNA fragments could be obtained from a highly processed medicinal product prepared from the skin of *Equus asinus* [26]. A recent report described using the ribosomal external transcribed spacer region to authenticate ginseng products such as tea and red ginseng extract [7]. However, how these products were processed and whether they contained ginseng residues was not mentioned.

Identification of herbal ingredients in decoctions is highly significant because this method will extend molecular authentication of herbal products. This method may also be useful for law enforcement authorities and may benefit consumers. Thus this study aims to evaluate the feasibility for molecular authentication methods by PCR and DNA sequencing for decoctions. We employed a single herb decoction with *P. ginseng* (“ginseng”) or *P. quinquefolius* (“American ginseng”), decoctions of two classical ginseng prescriptions with multiple herbal materials, and a commercial ready-to-serve ginseng soup to investigate the availability of DNA for molecular authentication. Then, we identified the DNA in the decoctions by multiplex PCR and DNA sequencing.

## Methods

### Samples

Herbal materials were purchased in a herbal shop (Zisun Pharmaceutical Ltd., Guangzhou, China). All herbal samples were identified with their organoleptic characteristics by Dr. David Tai-Wai Lau, Curator of the Shui-Ying Hu Herbarium, School of Life Sciences, The Chinese University of Hong Kong according to the *Pharmacopoeia of the People’s Republic of China* (2010 edition) [22] and confirmed by DNA sequencing. All authentic voucher specimens were deposited in the Institute of Chinese Medicine, The Chinese University of Hong Kong (Table 1).

**Table 1:** Herbal samples*

**Scientific name**	**Herbal material name**	**Method of processing**	**Place of collection**	**Voucher**
*Aconitum carmichaeli* Debx.	Aconiti Lateralis Radix Praeparata (*Fuzi*)	Dried, salted	Sichuan, China	ICM 2014-3452
*Atractylodes macrocephala* Koidz.	Atractylodis Macrocephalae Rhizoma (*Baizhu*)	Dried, sliced	Zhejiang, China	ICM 2014-3449
*Panax ginseng* C. A. Mey.	Ginseng Radix et Rhizoma (*Renshen*)	Dried	Jilin, China	ICM 2005-2750
				ICM 2005-2779
				ICM 2014-3448
*Glycyrrhiza uralensis* Fisch.	Glycyrrhizae Radix et Rhizoma (*Gancao*)	Dried, sliced	Inner Mongolia, China	ICM 2014-3450
*Panax quinquefolius* L.	Panacis Quinquefolii Radix (*Xiyangshen*)	Dried	USA	ICM 1713
				ICM 901276
*Zingiber officinale* Rosc.	Zingiberis Rhizoma (*Ganjiang*)	Dried, sliced	Sichuan, China	ICM 2014-3451

*All herbal samples were authenticated by Dr. David Tai-Wai Lau, Curator of the Shui-Ying Hu Herbarium, School of Life Sciences, The Chinese University of Hong Kong. In addition, DNA was extracted from the samples for DNA sequencing and results showed correct species identification.

Dust and soil on the surface of samples were removed by cleaning with water. These herbs were prepared for decoctions. A commercial ready-to-serve ginseng soup, Korean Ginseng Chicken Stew (Harim, Korea; product code: 8801492307002; voucher code: T3475), which claimed to contain *P. ginseng* on the label, was purchased from a local supermarket.

### Preparation of decoctions

A single herb decoction was prepared from *P. ginseng* or *P. quinquefolius*. Each herb was divided into two equal portions. One portion was pulverized into fine powder by a grinder. Another portion was retained in its sliced form. Herb (10 g) was boiled in 200 mL double-distilled water. Volume of the decoction was maintained at 200 mL by adding water. Herbal decoction was collected after 30, 60 and 120 min of boiling.

Two multi-herb decoctions (i) ginseng and prepared aconite, and (ii) ginseng and ginger combination were prepared. (i) was prepared from *Aconitum carmichaeli* and *P. ginseng* according to the protocol from *A Repertory of Traumatology. A. carmichaeli* (9 g) and *P. ginseng* (12 g) were cut into small pieces and boiled in 200 mL double-distilled water [27] for 60 min. (ii) was prepared from *Atractylodes macrocephala*, *P. ginseng*, *Glycyrrhiza uralensis* and *Zingiber officinale* according to the protocol from *Synopsis of Golden Chamber*. Each herb (46.9 g) was boiled for 180 min in 1600 mL double-distilled water until 600 mL decoction remained [28].

### Treatment of decoction samples

For each decoction sample collected at different times of boiling, large sediments were removed by MiniSpin centrifuge (Eppendorf, Hamburg, Germany). One portion of the supernatant was used for direct PCR and was regarded to be “crude DNA”. Another portion was lyophilized, followed by DNA extraction using a QIAquick Nucleotide Removal kit (Qiagen, Hilden, Germany) according to manufacturer instructions for the single herb decoctions; modified cetyltrimethylammonium bromide (CTAB) DNA extraction (protocol 1) (Additional file 1) for the multi-herb decoctions; and modified CTAB DNA extraction (protocol 2) (Additional file 1) for the Korean Ginseng Chicken Stew. This portion was regarded to be “extracted DNA”. Crude and extracted DNA were stored at −20°C immediately and PCR analyses were carried out within one month.

### Sequence alignment and primer design

Forty five DNA sequences of herbs were downloaded from the GenBank, National Center for Biotechnology Information (NCBI). These reference sequences were from *A. carmichaeli* trnH-psbA intergenic spacer, *A. macrocephala* ITS2, *G. uralensis* ITS2, *P. ginseng* and *P. quinquefolius* 26S-18S intergenic spacer and *Z. officinale* ITS2 (Additional file 2). These multi-copy sequences are abundant in the herbs, available in GenBank, highly variable and can be used effectively for identification.

Sequence alignment was carried out using ClustalW2 (European Bioinformatics Institute, Hinxton, UK). Primers were designed with sequences shown in Table 2. Their melting temperatures, percentage of GC content, hairpin formation, as well as formation of self-dimers and hetero-dimers were determined by OligoAnalysis v3.1 (Integrated DNA Technologies, Coralville, IA, USA).

**Table 2:** **Primers used for PCR amplification and sequencing**

**Herbal species**	**Primer pair**	**Primer name**	**Direction**	**Primer sequence (5′ to 3′)**	**Amplicon size (bp)**	**Annealing temperature (°C)**	**Gene or spacer region**
*A. carmichaeli*	1	Aca_F1	Forward	GGTGCATGTCTGGCTTAG	148	66	trnH-psbA
		Aca_R1	Reverse	TCACCAAACCAACACCAATC			
*A. macrocephala*	2	Ama_F1	Forward	CGACCCGCGAACATGTAAC	108	56	ITS2
		Ama_R1	Reverse	CGAGGCCCCGATAGGTG			
*G. uralensis*	3	Gur_F1	Forward	ACAGACCGTTGCCCGAC	106	57	ITS2
		Gur_R1	Reverse	CAGTTTTGAGCCAACCGTGAG			
*P. ginseng*	4	Pgi_F1	Forward	GGCAGTTGGCTAATGAAAGGTTGTA	88	64	26S-18S
		Panax_R1	Reverse	AAGCACCGCTATGCGCGAA			
	5	Pgi_F2	Forward	TGAAAGGTTGTAATAGTTT	249	48	26S-18S
		Pgi_R1	Reverse	AATGAAAAGGAATGAAAG			
	6	Pgi_F3	Forward	ACGGTTGCTTTTCCATCATTGTGT	311	64	26S-18S
		Panax_R1	Reverse	AAGCACCGCTATGCGCGAA			
	7	Pgi_SPF1	Forward	TGTCGGGCAAGGCCAAAAATG	121	64	26S-18S
		Panax_R1	Reverse	AAGCACCGCTATGCGCGAA			
	8	Panax_F1	Forward	GGTGCTTTGAGTGCTGCTGA	121 and 191	64	26S-18S
		Pgi_SPF1	Forward	TGTCGGGCAAGGCCAAAAATG			
		Panax_R1	Reverse	AAGCACCGCTATGCGCGAA			
*P. ginseng*, *P. quinquefolius*	9	Panax_F1	Forward	GGTGCTTTGAGTGCTGCTGA	191	64	26S-18S
		Panax_R1	Reverse	AAGCACCGCTATGCGCGAA			
*Z. officinale*	10	Zof_F1	Forward	GTAAAAGTCGGCAGTCGC	106	65	ITS2
		Zof_R1	Reverse	CACGCAGGGTCTCTTGAGG			

### PCR and DNA sequencing

Different DNA loci were amplified by conventional PCR or multiplex PCR using the designed primers (Table 2). For conventional PCR, a 25 μL reaction mixture comprising 1× PCR buffer (75 mM Tris, pH 8.8, 20 mM (NH_4_)_2_SO_4_, 1.5 mM MgCl_2_, 0.01% Tween 20), 0.2 mM each of deoxynucleotide triphosphates, 1.5 mM MgCl_2_, 0.4 μM of each primer, 10 ng DNA sample and 10 U/μL of *Taq* DNA polymerase was used. Multiplex PCR was set up with 0.3 μM forward conserved primer, 0.4 μM forward *P. ginseng-*specific primer and 0.5 μM reverse conserved primer (primer pair 8 in Table 2). Reactions were conducted by a Veriti Thermal Cycler (Applied Biosystems, Foster City, CA, USA) through 35 cycles of 95°C for 30 s, at the specific annealing temperature of each primer pair (Table 2) for 20 s and 72°C for 30 s. Amplicons were electrophoresed and visualized on 3% agarose gels stained with ethidium bromide. Amplicons were purified by a DNA Gel Extraction kit (Biomed, Beijing, China) followed by DNA sequencing (Beijing Genomics Institute, Hong Kong, China). Percentage maximum coverage and identity between amplified and reference DNA sequences were determined by the Basic Local Alignment Search Tool (BLAST) of NCBI.

### DNA concentration and statistical analyses

Extracted DNA was diluted so that its concentration was the same as that in the decoction. DNA concentration was measured using a Qubit 2.0 Fluorometer (Invitrogen, Carlsbad, CA, USA) and presented as the mean ± standard deviation. Differences between boiling times were assessed by one-way analysis of variance. Differences between sliced and pulverized samples at the same boiling time were analyzed by the two-sample *t* test. *P <* 0.05 was considered statistically significant.

## Results

### PCR products and DNA concentration in single herb decoction

**Figure 1:**
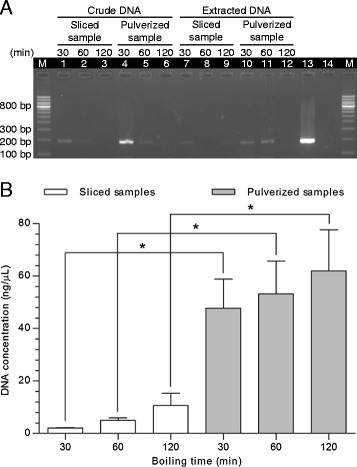
**PCR amplification and DNA concentration in decoction with sliced or pulverized sample at different times of boiling. (A)** *P. ginseng* sample was sliced (lanes 1–3, 7–9) or pulverized (lanes 4–6, 10–12) and boiled for 30 min (lanes 1, 4, 7, 10), 60 min (lanes 2, 5, 8, 11) and 120 min (lanes 3, 6, 9, 12). Crude DNA (lanes 1–6) and extracted DNA with concentration adjusted to that in the original decoction (lanes 7–12) were amplified by primer pair 9. Lane 13 is the positive control with DNA from *P. ginseng* and lane 14 is the negative control without DNA. Lane M represents the DNA size ladder. **(B)** The concentration of extracted *P. ginseng* DNA was adjusted to make it similar to that in the original decoction and measured by Qubit 2.0 Fluorometer. Water was used as blank and the data represent mean ± standard deviation (n = 3). Difference between boiling time was analyzed by one-way analysis of variance. Difference between sliced and pulverized samples at the same boiling time was analyzed by two sample *t* test (*p < 0.05).

We investigated if a 191 bp DNA of the *P. ginseng* 26S-18S intergenic spacer region could be amplified from the single herb *P. ginseng* decoction prepared by boiling sliced or pulverized samples for 30, 60 and 120 min. With the concentration of the crude and extracted DNA adjusted to that in the original decoction, visible PCR products were found in crude and extracted DNA with samples boiled for 30 min. More PCR product was obtained in pulverized samples boiled for 60 min (Figure 1A), and there was a significant increase in DNA released into the decoction in pulverized samples for each boiling time compared with sliced samples (30 min: *P* = 0.0280; 60 min: *P* = 0.0319; 120 min: *P* = 0.0479) (Figure 1B). A visible PCR product was not observed upon boiling for 120 min, although it was a time at which the highest DNA concentration was measured.

### Size of PCR product with boiling time

**Figure 2:**
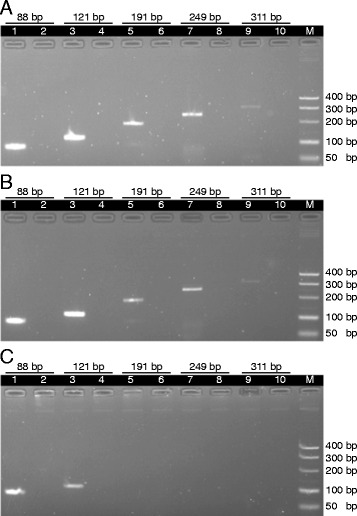
**DNA degradation with time of boiling.** Pulverized *P. ginseng* sample was boiled for **(A)** 30 min, **(B)** 60 min and **(C)** 120 min. The extracted DNA was amplified by PCR to produce 88 bp (lane 1) using primer pair 4, 121 bp (lane 3) using primer pair 7, 191 bp (lane 5) using primer pair 9, 249 bp (lane 7) using primer pair 5 and 311 bp (lane 9) using primer pair 6. Lanes 2, 4, 6, 8, 10 are negative controls of its previous lanes without decoction DNA. Lane M represents the DNA size ladder.

We investigated the relationship between boiling time and the size of amplifiable DNA. DNA fragments with sizes (in bp) of 88, 121, 191, 249 and 311 were amplified. For pulverized samples boiled for 30 and 60 min, DNA fragments of size 88-311 bp were amplified (Figure 2A and B). However, only short PCR products with sizes ranging from 88-121 bp were amplified for samples boiled for 120 min (Figure 2C).

### Multiplex PCR for differentiation between ginseng and American ginseng

**Figure 3:**
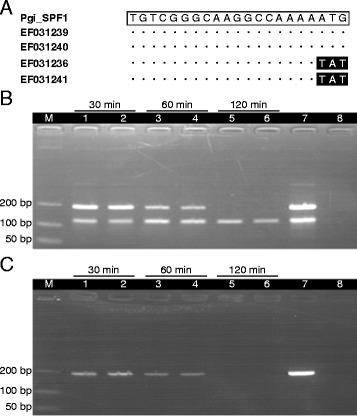
**Multiplex PCR in differentiation of ginseng and American ginseng. (A)** Nucleotide sequences of the forward *P. ginseng-*specific primer (Pgi_SPF1) with comparison to the aligned reference sequences of *P. ginseng* (Genbank accession number: EF031239, EF031240) and *P. quinquefolius* (Genbank accession number: EF031236, EF031241). Open box indicates the primer sequence, dots and closed boxes indicate nucleotides identical and different from the primer sequence, respectively. **(B** and **C)** Amplification of the extracted DNA from two vouchers of pulverized **(B)** *P. ginseng* (ICM 2005–2750, ICM 2005–2779) and **(C)** *P. quinquefolius* (ICM 1713, ICM 901276) samples by multiplex PCR using primer pair 8. Samples in herbal decoction were boiled for 30 min (lanes 1–2), 60 min (lanes 3–4) and 120 min (lanes 5–6), respectively. Lane 7 is the positive control with DNA from the concerned herbal sample and lane 8 is the negative control without DNA. Lane M represents the DNA size ladder.

Multiplex PCR was developed to differentiate *P. ginseng* from *P. quinquefolius*. PCR master mixes involved one pair of conserved primers for *P. ginseng* and *P. quinquefolius* (Panax_F1 and Panax_R1), and a forward *P. ginseng*-specific primer (Pgi_SPF1) region for pairing with the same reverse conserved primer (Panax_R1) (Additional file 3). Three nucleotides at the 3′ end of the 21 nucleotide forward *P. ginseng-*specific primer were different from those of the DNA sequence of *P. quinquefolius* (Figure 3A). As a result, the 191 bp amplicon was found in *P. ginseng* and *P. quinquefolius* decoction and the 121 bp amplicon was found only in the *P. ginseng* decoction (Figure 3B and C). However, the 191 bp amplicon was not found in the decoction boiled for 120 min (Figure 3B and C) due to DNA degradation (Figure 2C).

### Identification of herbs used in multi-herb decoctions and commercial soup

**Figure 4:**
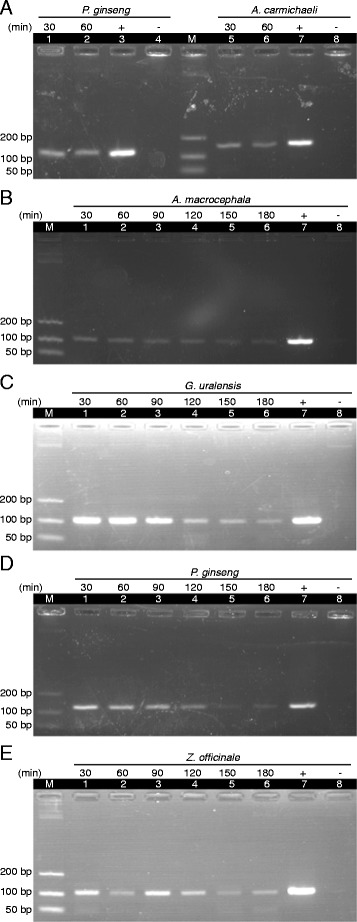
**PCR amplification of extracted DNA in the prescriptions. (A)** Two herbs decoction: Decoction of Ginseng and Prepared Aconite. The prescription was boiled for 30 min (lanes 1 and 5) and 60 min (lanes 2 and 6). Extracted DNA was amplified by primer pair 7 (lanes 1–2) for *P. ginseng* and primer pair 1 (lanes 5–6) for *A. carmichaeli*. Lanes 3 and 7 are positive controls with DNA from the concerned herbal sample and lanes 4 and 8 are negative controls without DNA. **(B-E)** Four herbs decoction: Ginseng and Ginger Combination. The prescription was boiled for 30 min (lane 1), 60 min (lane 2), 90 min (lane 3), 120 min (lane 4), 150 min (lane 5) and 180 min (lane 6). Primers specific for **(B)** *A. macrocephala* (Primer pair 2), **(C)** *G. uralensis* (Primer pair 3), **(D)** *P. ginseng* (Primer pair 7) and **(E)** *Z. officinale* (Primer pair 10) were used to amplify the decoction DNA. Lane 7 is positive control with DNA from the concerned herbal sample and lane 8 is negative control without DNA. Lane M represents the DNA size ladder.

**Figure 5:**
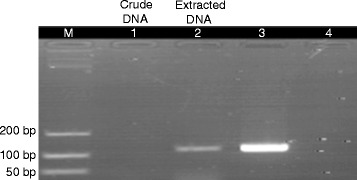
**PCR amplification of** ***P. ginseng*** **DNA in the commercial ginseng soup.** Crude (lane 1) and extracted DNA (lane 2) were amplified by primer pair 7. Lane 3 is the positive control with DNA from *P. ginseng* and lane 4 is the negative control without DNA. Lane M represents the DNA size ladder.

In a decoction involved two herbs (ginseng and prepared aconite), *A. carmichaeli* (148 bp) and *P. ginseng* (121 bp) could be amplified in extracted DNA with samples boiled for 30 and 60 min (Figure 4A). In another decoction involved four herbs (ginseng and ginger combination), *A. macrocephala* (108 bp), *G. uralensis* (106 bp), *P. ginseng* (121 bp) and *Z. officinale* (106 bp) were amplified in extracted DNA with samples boiled for 180 min (Figure 4B-E). We could also amplify the *P. ginseng* component in Korean Ginseng Chicken Stew after DNA was extracted (Figure 5).

## Discussion

In the present study, DNA released into the decoction by boiling could be amplified by PCR. BLAST analyses of these amplicons showed ≥99% coverage and identity between the amplified and reference sequences (Additional file 4).

Pulverization of the sample increased the yield of DNA released into the decoction (Figure 1B) due to the increase in the surface area of the sample. The highest DNA concentration was measured in the decoction boiled for 120 min but a PCR product was not observed (Figure 1A). An increase in the duration of heat treatment can cause a higher degree of DNA fragmentation in meat samples [29]. This phenomenon might also be the case in decoction, resulting in a less amplifiable DNA template that is intact. We confirmed this hypothesis by amplifying DNA of sizes ranging from 88-311 bp. Only short PCR products of ≤121 bp were observed with 120 min boiling (Figure 2C). Therefore, the amplicon to be studied in the decoction should be short. Sometimes this requirement may mean that the ability to differentiate between species is sacrificed. In our case, because of limited sequences in GenBank, our primers designed for *A. carmichaeli* (primer pair 1 in Table 2) and *G. uralensis* (primer pair 3 in Table 2) were specific at the genus level. Other primers had specificity at the species level (Additional file 4).

The PCR product was detected by amplifying crude DNA in the single herb decoction directly (Figure 1A). The chemicals released from herbs might not be detrimental to PCR in certain cases. This phenomenon increased the efficiency of authentication because time-consuming DNA extraction procedures could be omitted.

In the market, it is common to find cultivated *P. ginseng* as an adulterant of *P. quinquefolius*. These two herbs have different pharmacological effects and chemical compositions [30,31]. We investigated the possibility of differentiating *P. ginseng* and *P. quinquefolius* in decoctions by multiplex PCR. PCR master mixes involved one pair of conserved primers for *P. ginseng* and *P. quinquefolius*, and a forward *P. ginseng*-specific primer (Additional file 3). The 191 bp conserved amplicon was amplified and served as the positive control to ensure sufficient intact DNA fragments presented in the decoction. There were three nucleotide mismatches with the DNA sequence of *P. quinquefolius* at the 3′ end of the forward *P. ginseng*-specific primer sequence (*i.e.*, nearly 15% mismatch) (Figure 3A), which made the primer sufficiently specific for producing the 121 bp *P. ginseng*-specific PCR product (Figure 3B). This approach may be explored for differentiating between genuine and adulterant herbs in a decoction and, if coupled to quantitative PCR, for finding the ratio of the two herbs.

Herbal prescriptions usually consist of more than one herb. Two classical ginseng prescriptions (ginseng and prepared aconite, ginseng and ginger combination) and a commercial ginseng soup were tested to illustrate the possibility of identification of herbs by DNA markers in multi-herb decoctions. Results showed that DNA fragments of the relevant herbal ingredients were amplified (Figures 4 and 5).

Some of the herbs used in the prescriptions were dried and some were even processed (Table 1). In comparison with fresh materials, carrying out molecular authentication for dried and processed herbal materials is difficult because DNA is degraded or interacts with other chemicals. Several protocols have been developed to facilitate DNA extraction of different herbal samples: silica binding [32], salting-out precipitation [33], magnetic beads binding, such as ChargeSwitch gDNA Plant Kit (Invitrogen, Carlsbad, CA, USA) and anion exchange purification, such as Genomic Tip 20/G (Qiagen, Hilden, Germany) [34]. There are also reports to extract PCR amplifiable DNA from maize, soy bean [35], fish derived food products [36] and rice wine [37]. Our study shows the presence of PCR amplifiable DNA fragments of herbal materials in the aqueous solution after boiling. This phenomenon may be investigated further for other herbal decoctions.

## Conclusions

DNA could be amplified from extensively boiled ginseng decoctions, multi-herb decoctions and commercial soup although DNA degradation was critical to successful PCR.

## Additional files

Additional file 1:
**Modified CTAB extraction protocols.**
Additional file 2:
**GenBank accession numbers of herbal species for primer design.**
Additional file 3:
**Comparison of the aligned 26S-18S sequences of** ***P. ginseng*** **and** ***P. quinquefolius*** **for multiplex PCR primer design.**
Additional file 4:
**DNA sequences of amplicons and its percentage coverage and identity to NCBI GenBank DNA sequences.**

## Abbreviations

BLAST: Basic Local Alignment Search Tool
CTAB: cetyltrimethylammonium bromide
DNA: Deoxyribonucleic acid
ITS2: Ribosomal internal transcribed spacer 2
NCBI: National Center for Biotechnology Information
PCR: Polymerase chain reaction
