# Supplementary material for: Identification of constituent herbs in ginseng decoctions by DNA markers
Source: Chin Med. 2015 Jan 30;10(1):1. doi: 10.1186/s13020-015-0029-x (PMC4318153; doi:10.1186/s13020-015-0029-x)
Supplement: Additional file 1: — Modified CTAB extraction protocols. [file 13020_2015_29_MOESM1_ESM.doc]

**Modified CTAB extraction protocols [1]**

Modified CTAB extraction (Protocol 1): For the two multi-herb decoctions

- Place 1 mL of decoction in a microcentrifuge tube (1.5 mL) and lyophilize.
- Add 400 μL of extraction buffer^1^.
- Add 400 μL of CTAB solution (2%)^2^ and place it on ice for 5 min.
- Centrifuge at 12,100 x *g* in a microcentrifuge^3^ at 4^o^C for 5 min and transfer the supernatant to a new tube.
- Gently extract with equal volume of chloroform: isoamyl alcohol (24: 1), centrifuge at 12,100 x *g* for 10 min and transfer the supernatant to a new tube.
- Repeat the above step until there are no precipitates at the bottom of the aqueous phase.
- Add 2/3 volume isopropanol and incubate the tube at room temperature for 10 min to precipitate the DNA.
- Centrifuge at 12,100 x *g* for 5 min, remove supernatant, and wash the DNA pellet with 70% ethanol, air dry, and resuspend in 50 μL water.

Modified CTAB extraction (Protocol 2): For ‘Korean Ginseng Chicken Stew’

- Place 1 mL of soup in a microcentrifuge tube (1.5 ml) and lyophilize.
- Add 400 μL of extraction buffer^1^ containing proteinase K (50 µg), incubate at 37^o^C for 1 h.
- Add 400 μL of CTAB solution (2%)^2^ and place it on ice for 5 min.
- Centrifuge at 12,100 x *g* in a microcentrifuge^3^ at 4^o^C for 5 min and transfer the supernatant to a new tube.
- Gently extract with equal volume of phenol: chloroform: isoamyl alcohol (25: 24: 1), centrifuge at 12,100 x *g* for 10 min and transfer the supernatant to a new tube.
- Repeat the above step until there are no precipitates at the bottom of the aqueous phase.
- Gently extract with equal volume of chloroform: isoamyl alcohol (24:1) once, centrifuge at 12,100 x *g* for 10 min and transfer the supernatant to a new tube.
- Add 2/3 volume isopropanol and incubate the tube at room temperature for 10 min to precipitate the DNA.
- Centrifuge at 12,100 x *g* for 5 min, remove supernatant, and wash the DNA pellet with 70% ethanol, air dry, and resuspend in 50 μL water.

**Notes**

1. Extraction buffer: 200 mM Tris-HCl (pH 8.0), 200 mM NaCl, 25 mM EDTA, 0.5% SDS.
2. 2% CTAB (cetyltrimethylammonium bromide) solution: 2% CTAB (w/v), 100 mM Tris-HCl (pH 8.0), 20 mM EDTA (pH 8.0), 1.4 M NaCl, 1% PVP (polyvinylpyrrolidone) Mr 40,000.
3. Microcentrifuge machine used: MiniSpin (Eppendorf, Hamburg, Germany).

**References**

1. Kang H, Cho Y, Yoon U, Eun M: **A Rapid DNA Extraction Method for RFLP and PCR Analysis from a Single Dry Seed**. *Plant Mol Biol Rep* 1998, **16:**1-9.
