# Supplementary material for: Identification of constituent herbs in ginseng decoctions by DNA markers
Source: Chin Med. 2015 Jan 30;10(1):1. doi: 10.1186/s13020-015-0029-x (PMC4318153; doi:10.1186/s13020-015-0029-x)
Supplement: Additional file 2: — GenBank accession numbers of herbal species for primer design. [file 13020_2015_29_MOESM2_ESM.doc]

**GenBank accession numbers of herbal species for primer design**

| **Herbal species** | **Gene or spacer region** | **GenBank accession number*** |
| --- | --- | --- |
| *A. carmichaeli* | trnH-psbA | FJ821166, GQ337748, GQ337765, GQ337766, GQ337767, GQ337769, GQ337770, GQ337771, GQ337773, GQ337823, GQ337852, GQ337853 |
| *A. macrocephala* | ITS2 | AB219406, JX083751, JX083752, JX083754, JX083755, JX083756 |
| *G. uralensis* | ITS2 | AB280738, AB649775, EU418258, HQ229003, JF421503, JF421504 |
| *P. ginseng* | 26S-18S | EF031235, EF031237, EF031238, EF031239, EF031240, EU232122, EU232123, EU232124, EU232125, EU232126, KF680553 |
| *P. quinquefolius* | 26S-18S | EF031236, EF031241 |
| *Z. officinale* | ITS2 | AM777878, DQ064590, GQ434445, GQ434446, GU180375, GU180376, KC582873, KF694808 |

* The GenBank published DNA sequences are identical to the DNA sequences of our reference species
