# Supplementary material for: Identification of constituent herbs in ginseng decoctions by DNA markers
Source: Chin Med. 2015 Jan 30;10(1):1. doi: 10.1186/s13020-015-0029-x (PMC4318153; doi:10.1186/s13020-015-0029-x)
Supplement: Additional file 3: — Comparison of the aligned 26S-18S sequences of P. ginseng and P. quinquefolius for multiplex PCR primer design. [file 13020_2015_29_MOESM3_ESM.doc]

**Comparison of the aligned 26S-18S sequences of *P. ginseng* and *P. quinquefolius* for multiplex PCR primer design**


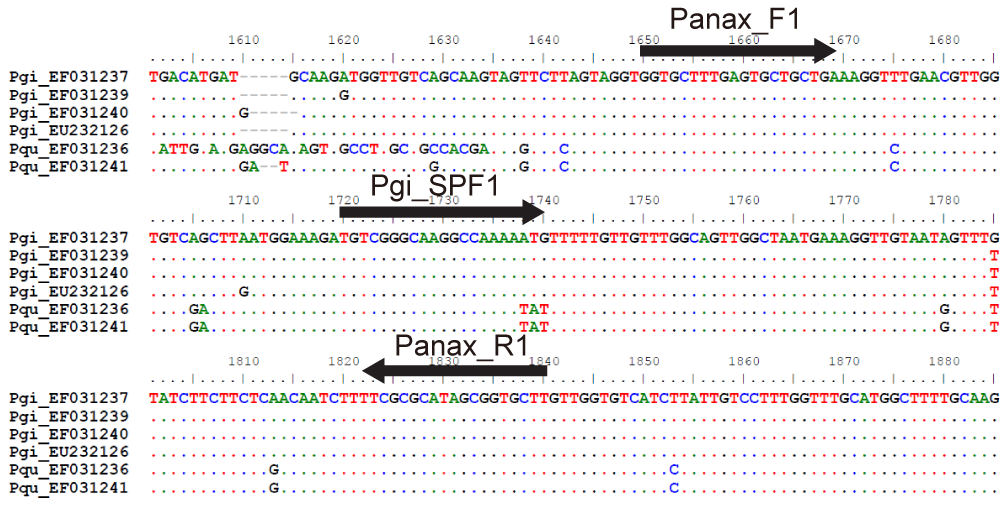


GenBank accession number with prefix “Pgi” represents *P. ginseng* sequences; “Pqu” represents *P. quinquefolius* sequences. Primers Panax_F1 and Panax_R1 amplified both two species DNA while primers Pgi_SPF1 and Panax_R1 amplified *P. ginseng* DNA only. Therefore only 1 amplicon is produced for *P. quinquefolius* sample while 2 amplicons are produced for *P. ginseng* sample.
