# Supplementary material for: Identification of constituent herbs in ginseng decoctions by DNA markers
Source: Chin Med. 2015 Jan 30;10(1):1. doi: 10.1186/s13020-015-0029-x (PMC4318153; doi:10.1186/s13020-015-0029-x)
Supplement: Additional file 4: — DNA sequences of amplicons and its percentage coverage and identity to NCBI GenBank DNA sequences. [file 13020_2015_29_MOESM4_ESM.doc]

**DNA sequences of amplicons and its percentage coverage and identity to NCBI GenBank DNA sequences**

| **Herbal species** | **Primer pair** | **Gene or spacer region** | **DNA sequence (Excluding primer sequence)** | **BLAST result**  **(matched/ total available sequence)** | **% coverage** | **% identity** |
| --- | --- | --- | --- | --- | --- | --- |
| *A. carmichaeli* | 1* | trnH-psbA | TGTATATGAGTCATTGAAGTTGCAGGAGCAATACCCAGCCTCTTAACAGAACAAGAAATTGGGTATTGCTCCTGCATTTTTTTGTATTAAGTAAAAATTTGACTTTAACA | *A. carmichaelii* (8/13)  *A. kusnezoffii* (3/13)  *A. soongaricum* (2/13) | 100%  100%  100% | 99%~100%  100%  100% |
| *A. macrocephala* | 2 | ITS2 | GACAACCGGGCATCGGGGGGAACGGGCGCAAGCCCGGGACCTGCGGCGCTCAGTCGGCGAGCGTCCGTGGCG | *A. macrocephala* (6/6) | 100% | 100% |
| *G. uralensis* | 3* | ITS2 | GCCAATTGCCTCGCGATAGGTACTTTGGTTGTGCAGGGTGAATGTTGGCTTCCCGTGAGCATTGCGGC | *G. uralensis* (6/8)  *Glycyrrhiza sp.* (1/8)  G. aspera (1/8) | 100%  100%  100% | 100%  100%  100% |
| *P. ginseng* | 4 | 26S-18S | ATAGTTTTGAAATTATGTTTTCTTATCTTCTTCTCAACAATCTT | *P. ginseng* (8/8) | 100% | 100% |
|  | 5 | 26S-18S | GAAATTATGTTTTCTTATCTTCTTCTCAACAATCTTTTCGCGCATAGCGGTGCTTGTTGGTGTCATCTTATTGTCCTTTGGTTTGCATGGCTTTTGCAAGTCGTGTGAGTTGGGGTGCATTAGATGGCGTTGGCATAGTGTACGTATGGCGCGTGAGTGGTGTTTGGTTTGTTTGGGTGGGTTGGATCCCTGCTTGTGCAGCGACGACCGCT | *P. ginseng* (11/11) | 100% | 100% |
|  | 6 | 26S-18S | TGGCTTGAGGAAATCGAGCACCTGGTTGAGGGCCCTTCTTGACATTTTATTGACATGATGCAAGATGGTTGTCAGCAAGTAGTTCTTAGTAGGTGGTGCTTTGAGTGCTGCTGAAAGGTTTGAACGTTGGTGGCCTATGCTGATGTGTCAGCTTAATGGAAAGATGTCGGGCAAGGCCAAAAATGTTTTTGTTGTTTGGCAGTTGGCTAATGAAAGGTTGTAATAGTTTTGAAATTATGTTTTCTTATCTTCTTCTCAACAATCTT | *P. ginseng* (4/4) | 100% | 99% |
|  | 7 | 26S-18S | TTTTTGTTGTTTGGCAGTTGGCTAATGAAAGGTTGTAATAGTTTTGAAATTATGTTTTCTTATCTTCTTCTCAACAGTCTT | *P. ginseng* (5/5) | 100% | 99% |
| *P. ginseng* | 9 | 26S-18S | AAGGTTTGAACGTTGGTGGCCTATGCTGATGTGTCAGCTTAATGGAAAGATGTCGGGCAAGGCCAAAAATGTTTTTGTTGTTTGGCAGTTGGCTAATGAAAGGTTGTAATAGTTTTGAAATTATGTTTTCTTATCTTCTTCTCAACAATCTT | *P. ginseng* (6/6) | 100% | 99%~100% |
| *P. quinquefolius* | 9 | 26S-18S | AAGGTCTGAACGTTGGTGGCCTATGCTGATGTGTCGACTTAATGGAAAGATGTCGGGCAAGGCCAAAATATTTTTTGTTGTTTGGCAGTTGGCTAATGAAAGGTTGTAATGGTTTTGAAATTGTGTTTTCTTATCTTCTTCTCGACAATCTT | *P. quinquefolius* (2/2) | 100% | 100% |
| *Z. officinale* | 10 | ITS2 | GGGGCACGACGGGTGTTGGTCGCCGTGAGCGGGAACAGGACGTCGTCCCCGTCGTTTTCGGACTGAATT | *Z. officinale* (8/8) | 100% | 99%~100% |

* Primer specific to genus level
